# Pharmacokinetic Study of Anti-osteoarthritic Compounds of a Standardized Fraction from *Sphaeralcea Angustifolia*

**DOI:** 10.3390/ph14070610

**Published:** 2021-06-25

**Authors:** Jade Serrano-Román, Pilar Nicasio-Torres, Elizabeth Hernández-Pérez, Enrique Jiménez-Ferrer

**Affiliations:** 1 Centro de Investigación Biomédica del Sur (CIBIS), Instituto Mexicano del Seguro Social (IMSS), Argentina 1, Centro, Xochitepec CP 62790, Mexico; jadita_9@hotmail.com (J.S.-R.); pisaliva@yahoo.com.mx (P.N.-T.); 2 Doctorado en Ciencias Biológicas y de la Salud, División de Ciencias Biológicas y de la Salud, Universidad Autónoma Metropolitana Unidad Iztapalapa (UAM-Iztapalapa). San Rafael Atlixco 186, Vicentina, Iztapalapa, Ciudad de Mexico CP 09340, Mexico; sila@xanum.uam.mx

**Keywords:** pharmacokinetic, osteoarthritis, coumarins, scopoletin, tomentin, sphaeralcic acid, immunomodulation

## Abstract

*Sphaeralcea angustifolia* has been widely used in inflammatory conditions such as blows, bruises, fractures, and wounds. The compounds identified as active in plants and suspension cell culture of *S. angustifolia* were tomentin, scopoletin, and sphaeralcic acid. To consolidate the integral use of knowledge about the *S. angunstifolia* and strengthen its pharmacological use in patients with knee osteoarthritis, the pharmacokinetic behavior of the active compounds was characterized. The SaTSS (*S. angustifoloia* standardized in Tomentin, Scopoletin, and Sphaeralcic acid) anti-ostearthritic fraction was obtained from cell suspension. The analytical method of High-Performance Liquid Chromatography (HPLC) for tomentin, scopoletin, and sphaeralcic acid were validated determining the accuracy, precision linearity, sensibility, specificity, detection limits, and quantification time-range parameters, as well as extraction efficiency and stability of compounds. The pharmacokinetic assay was performed with ICR mice strain, in which the mice were administrated with a single oral or intravenous dose (400 mg/kg with 7.1 mg/kg of scopoletin and tomentin in mixture and 34.6 mg/kg of sphaeralcic acid) of the SaTSS standardized active fraction. The results of the validated analytical methods allowed establishing, in a validated manner, that a coumarin mixture and sphaeralcic acid present in the SaTES fraction were detected in plasma. According to the values of Akaike Information Criteria (AIC), Sum of Squares (SS), Schwarz Criteria (SC), and by the determination coefficient (*R*^2^), the compounds follow a two-compartment model.

## 1. Introduction

Osteoarthritis (OA) is the most common arthritis with a global prevalence of OA of 16.0% in individuals aged 15 and over, and of 22.9% in individuals aged 40 and over [1] constituted mainly by women [2]. The articular inflammation, regardless of its etiology, is associated with high direct and indirect costs, and represents the main cause of permanent disability in the right-holder population of the Instituto Mexicano del Seguro Social (IMSS, the institution with the largest coverage of health care in Mexico), which represents 11% of the consultations of family medicine in the first level of attention [3]. OA is a chronic degenerative disease with disabling consequences [2,4]. The treatment of OA is based on non-steroidal anti-inflammatories and glucocorticoids, as well as on biological and non-biological drugs that modify the disease; prolonged use of these treatments has adverse side effects [5]. There is not a relevant treatment available, therefore there is a need to find new drugs that have fewer side effects than those currently used; medicinal plants are a potential source to find new active compounds.

**Figure 1:**
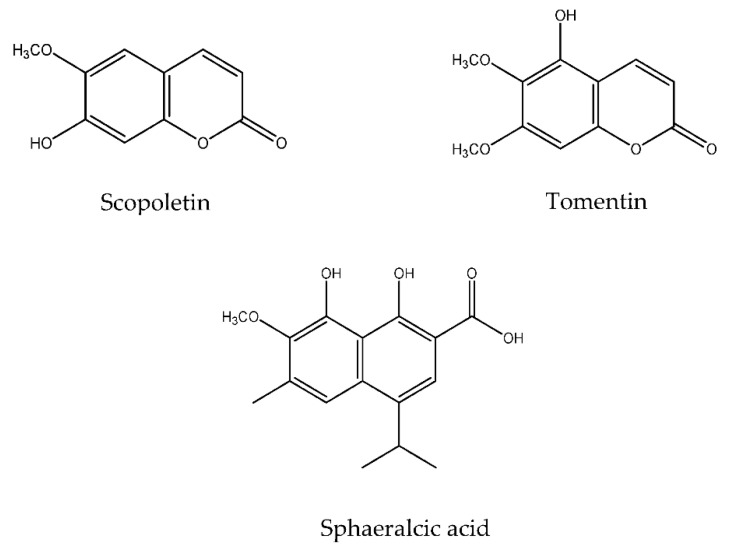
Chemical structures of active compounds standardized in the SaTSS fraction obtained from the *Sphaeralcea angustifolia* cell suspension [15].

*Sphaeralcea angustifolia* (Cav.) G. Don (Malvaceae) is used in Mexican traditional medicine to treat illnesses that involve an inflammatory process [6]. The dichloromethane extract from the aerial tissues of this species was active in acute inflammation and induced arthritis in murine models [7,8]. Dichloromethane extract administration modulates the response of pro- and anti-inflammatory interleukins in an arthritis-induced model in rats with complete Freund’s adjuvant [8,9]. A gel prepared with the dichloromethane extract of *S. angustifolia* aerial tissues standardized in scopoletin demonstrated therapeutic effectiveness and tolerability, capable of reducing the associated symptoms: Pain, inflammation, and joint stiffness in treating patients with hand osteoarthritis [10]. Furthermore, scopoletin also downregulated the overexpression of vascular endothelial growth factor, basic fibroblast growth factor, and interleukins in an adjuvant-induced arthritic model in rats; then, scopoletin ameliorated synovial hyperplasia, reduced the presence of inflammatory cells in the synovium, and diminished erosive changes in the cartilage and bone [11]. *S. angustifolia* has normative restriction [12] for its collection in its natural habitat; therefore, to obtain the phytopharmaceuticals responsible of the anti-osteoarthritis activity, the sustainable production of scopoletin, tomentin, and sphaeralcic acid (Figure 1) in the cell suspension cultivated in a stirred tank bioreactor was implemented [13]. It should be highlighted that the active compounds significantly increased anti-inflammatory interleukin responses in rats with experimental arthritis [11,14,15]. Derived from the advances mentioned above, in the development of anti-osteoarthritic phytomedicine formulated with standardized *Spheralceae angustifolia* extract, and which may be of medical interest to drug developers, the aim of this research was to determine the plasma concentrations of scopoletin, tomentin, and sphaeralcic acid present in the standardized fraction (SaTSS) from an *S. angustifolia* cell after a single oral or intravenous dose in mice.

## 2. Results

### 2.1. Chromatographic Profiling of Tomentin, Scopoletin, Sphaeralcic Acid, and SaTSS Standardized Fraction

**Figure 2:**
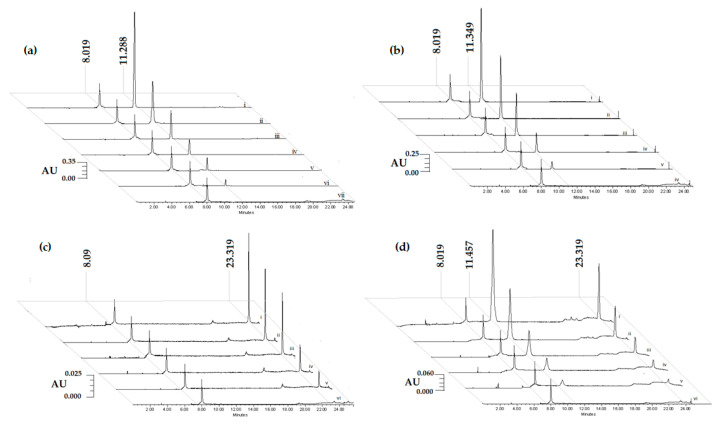
Chromatographic profiles of the concentration curves of tomentin (**a**), scopoletin (**b**), and sphaeralcic acid (**c**) pure active compounds, and SaTSS standardized fraction (**d**) obtained from *Sphaeralcea angustifolia* cell suspensions with the internal dopamine standard (rt = 8.019 min).

Figure 2 shows the chromatograms of standard dilutions from the highest to the lowest concentration (i to v or vi) for calibration curves of tomentin (Figure 2a) rt = 11.288 min (20, 10, 5, 2.5, 1.25, and 0.625 µg/mL), scopoletin (Figure 2b) rt = 11.349 min (20, 10, 5, 2.5, and 1.25 µg/mL), sphaeralcic acid (Figure 2c) rt = 23.319 min (40, 20, 10, 5, and 2.5 µg/mL), and SaTSS standardized fraction (Figure 2d) obtained from the CH_2_Cl_2_:CH_3_OH extract of *S. angustifolia* cell suspension (400, 200, 100, 50, and 25 µg/mL). When the SaTSS fraction was analyzed, a single peak was observed, consisting of the mixture of tomentin and scopoletin with a rt = 11.457 min, then the SaTSS fraction was standardized in a mixture of tomentin and scopoletin 17.19 mg/g, and sphaeralcic acid 86.4 mg/g. The internal standard used was dopamine rt = 8.019 min (10 µg/mL). Chromatograms vii (Figure 2a) and vi (Figure 2b–d) correspond to plasma samples without the compound or SaTSS fraction.

### 2.2. Validation Methods

#### 2.2.1. Standardization of Chromatographic Process

The methods were validated using the following criteria: For linearity and sensitivity, the data of tomentin were found to be linear along a concentration range of 0.62–20 µg/mL, scopoletin at 1.25–20 µg/mL, and sphaeralcic acid at 2.5–40 μg/mL. The regression equation for tomentin was (y) = 162,940(x) + 1268.2, r^2^ = 0.9994; for scopoletin, it was (y) = 165,407(x) + 16,720, r^2^ = 0.9993, and for sphaeralcic acid, it was (y) = 7381.9(x) + 1362.2, r^2^ = 0.9998, with a r^2^ > 0.99 indicating good linearity. Limits of Detection (LOD) and Limits of Quantification (LOQ) were calculated by the equations LOD = 3.3σ/s; LOQ = 10σ s (where s is the slope of the calibration curve and σ is the SD of the slope). The LOD and LOQ for tomentin were 0.068 and 0.207 μg/mL, with a precision value of Relative Standard Deviation (RSD) of 2.07% (Table S1, Supplementary Materials and Table 1), while for scopoletin these were 0.139 and 0.421 µg/mL with RSD 4.21%, and for sphaeralcic acid, 0.059 and 0.180 µg/mL with RSD 1.80%. RSD values < 15% were considered acceptable.

Under these chromatographic conditions, the number of theoretical plates of the column was between 26,700 < N > 18,760 for the active compounds, which was acceptable in terms of separation efficiency.

No interference was observed between plasma constituents with the SaTSS standardized fraction rich in tomentin, scopoletin, and sphaeralcic acid after extraction with acetonitrile:TFA 20% (*w/v*) 1:1. The active compounds and the internal standard were distinguished by comparing the chromatograms with the blank plasma chromatogram (Sample vii Figure 2a, and sample vi, Figure 2b–d).

With respect to procedure specificity, dopamine with a rt = 8.019 min was used as an internal standard for the determination of active compounds (Figure 2). Tomentin, scopoletin, and sphaeralcic acid showed good separation with respect to the internal standard (Figure 2a–c). In the SaTSS standardized fraction, the mixture of tomentin and scopoletin (rt = 11.457 min) and sphaeralcic acid were notable (Figure 2d). 

Intra- and inter-assay analyzed concentrations for tomentin and scopoletin were 2.5, 5, 10, and 20 µg/mL, while those for sphaeralcic acid were 2.5, 5, 10, 20, and 40 µg/mL. Accuracy (% Bias) = [(Cobs − Cnom)/Cnom] × 100 and precision (Relative Standard Deviation [RSD]) calculated from the observed concentrations is as follows: RSD = [Standard Deviation (SD)/Cobs] × 100 were calculated. Thus, when the values of the accuracy (BIAS) and precision (RSD) determination fall within a range of ± 15%, they were considered to be acceptable (Table 1).

#### 2.2.2. Standardization of the Extraction Process

For the first time, to our knowledge, a simultaneous and individual plasma extraction technique was reported for the three active compounds: Tomentin, scopoletin, and sphaeralcic acid. The extraction efficiency (Table S2, Supplementary Materials) of the active compounds present in the SaTES standardized fraction was determined by an inter-day analysis (*n* = 5). Compound concentrations extracted from plasma were 2.5, 5, 10, and 20 μg/mL for tomentin and scopoletin, and 2.5, 5, 10, 20, and 40 µg/mL for sphaeralcic acid. Recovery was determined with the peak area of each active compound, contrasted with the peak area of each extracted compound added to the plasma at the same concentration. The variation coefficients of the compound analyses were lower than 3.09%, indicating adequate recovery capacity of compounds during the sample extraction process. The extraction percentage on average was >96% in all concentrations of the active compounds analyzed (Table S2, Supplementary Materials).

According to the stability analyses of tomentin, scopoletin, and sphaeralcic acid present in the SaTSS standardized fraction (Table 2), the precision of samples preserved at room temperature was maintained within a range between 1.53 and 5.97%, and accuracy ranged from to −9.98 to 7.61%. Additionally, the active compounds were also stable when stored at −70 °C for at least 1 month; precision varied between 0.79 and 6.71%, and accuracy ranged from −7.1 to 7.61%. The results suggest that the tomentin, scopoletin, and sphaeralcic acid present in the SaTSS standardized fraction could be adequately analyzed due to sample stability in the storage processes to which the real samples from experimental animals would be subjected.

### 2.3. Plasma Level of Active Compounds

**Figure 3:**
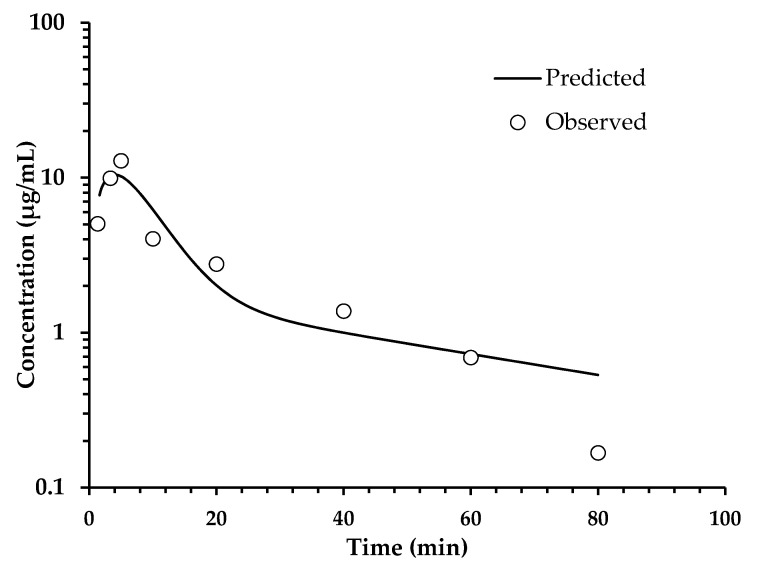
Analysis of variation of the sphaeralcic-acid concentrations in plasma with respect to the time in minutes, after oral administration (400 mg/kg) of the SaTSS standardized fraction. The line represents the prediction of the behavior of the concentration following a two-compartment model.

**Figure 4:**
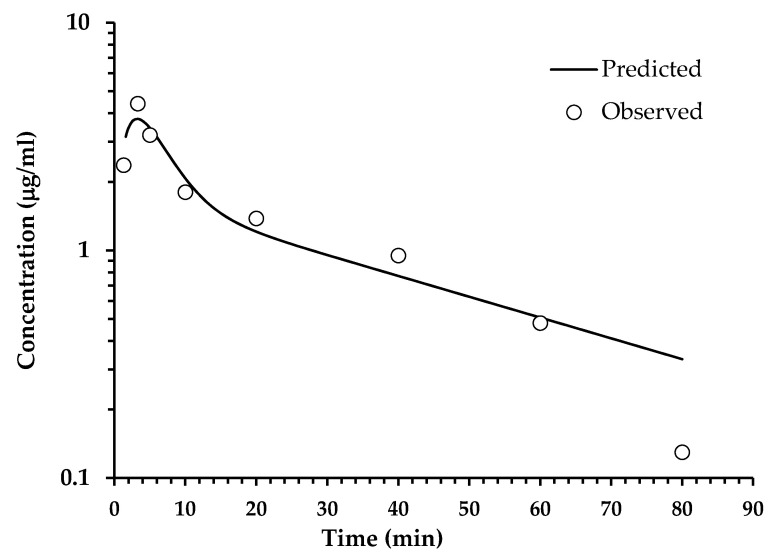
Analysis of variation of the tomentin and scopoletin (coumarin mixture) concentration in plasma with respect to time, after oral administration (400 mg/kg) of the SaTSS standardized fraction. The line represents the prediction of the behavior of the concentration following a two-compartment model.

**Figure 5:**
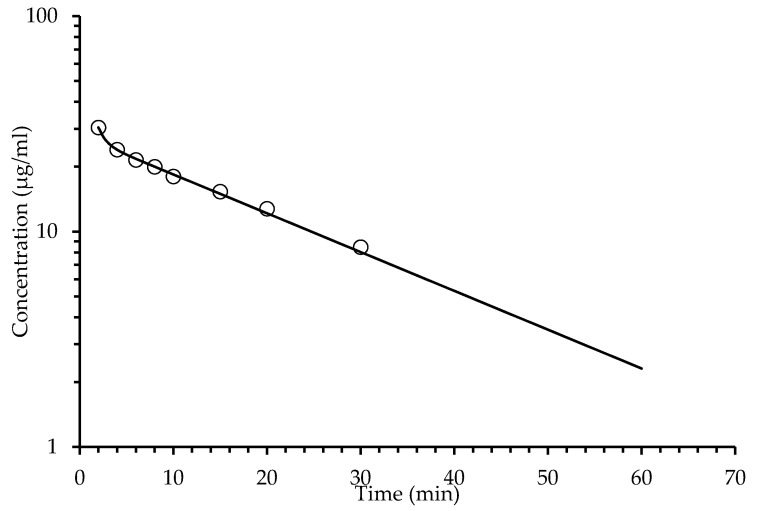
Analysis of variation of the sphaeralcic acid concentrations in plasma with respect to the time in minutes, after intravenous administration (400 mg/kg) of the SaTSS standardized fraction.

**Figure 6:**
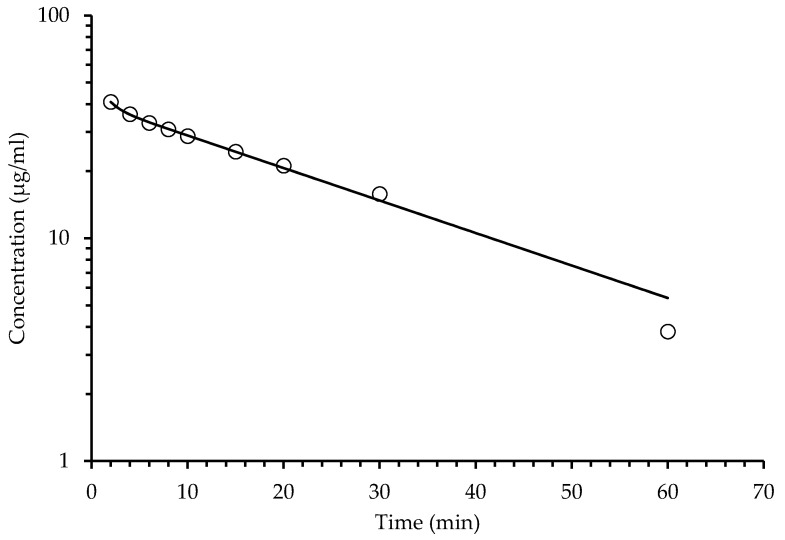
Analysis of variation of the tomentin and scopoletin (coumarin mixture) concentration in plasma with respect to time, after intravenous administration (400 mg/kg) of the SaTSS standardized fraction.

Determinations of HPLC (Table 3) from extracted plasma after a single oral administration of the SaTSS standardized fraction (400 mg/kg with 7.1 mg/kg of scopoletin and tomentin in mixture and 34.6 mg/kg of sphaeralcic acid) were performed at different times and expressed as the quantification mean of the mouse group (*n* = 10). The quantification of tomentin and scopoletin was given as a single value, since it was not possible to solve the chromatographic peaks obtained. The mixture of coumarins reached a maximum concentration at 3.30 min, showing a rapid decay, since, at min 80, the concentration fell below LOQ, and after this time, the coumarins were not detected in the plasma samples. In the same way, sphaeralcic acid showed its highest concentration in the quantification performed at minute five, with the plasma concentration falling below LOQ at min 80 (Figure 3 and Figure 4). In intravenous administration, the plasma concentration of the active compounds is observed from 2 to 60 min (Figure 5 and Figure 6).

### 2.4. Pharmacokinetic Assay 

#### 2.4.1. Pharmacokinetic Oral Administration 

The data (Table 4 and Table 5) were analyzed with the PKSolver program [16]. The graph constructed from the data of the behavior of the concentration versus the independent variable time established that the data fit a model with two compartments (Table 4) Then, the calculated pharmacokinetic parameters are mentioned first for the sphaeralcic acid and then for the mixture of coumarins.

After administration of the SaTES fraction, the compounds are found in higher concentrations in the central compartment (A), 413 µg/mL of spahaeralcic acid and 71.47 µg/mL for the coumarin mixture. The apparent first-order absorption rate constants (*k_a_*) were 0.27 and 0.39 min^−1^, in the same order as the previous one. The apparent first order elimination rate constants (*k*_10_) were 0.13 and 0.08 min^−1^, respectively. The mass transfer from the central compartment to the peripheral compartment presented an apparent first-order velocity constant (*k*_12_) of 0.114 min^−1^ for the spahaeralcic acid, which was lower than that observed for the coumarin mixture 0.201 min^−1^. In the opposite direction of the mass transfer, the apparent first-order velocity constant for the passage from the peripheral to the central compartment (*k*_21_) was 0.031 min^−1^ and 0.086 min^−1^ in the same order mentioned for *k*_12_. The mean lifetime in the central compartment (t_½_α) was 2.70 min and 1.96 min, less than in the peripheral compartment (t_½_β) where it was 44.65 min and 32.95 min and related to the absorption rate constant (t_½_ *k*_a_) 2.53 min y 1.77 min, in the same order as the previous ones. The values of the volume of distribution (V) and the clearance (CL) with respect to the bioavailability constant (F) were: 1.31 y 0.80 (mg)·(µg/mL)^−1^ for V and 0.17 y 0.07 (mg)/(µg/mL)/min for CL, keeping the same order as the previous ones. The values of the maximum time (T_max_) and maximum concentration (C_max_) of 4.00 min and 3.23 min for the first parameter and 10.44 µg/mL and 3.77 µg/mL of the second parameter are also reported, in the same order. The areas under the curve were calculated for the defined start of the interval (0 min) at the end of the evaluation (240 min) and for the infinite dilution (0 → ∞), obtaining the following values: 173.72 and 84.20 (µg/mL·min^−1^) for the first of the parameters and 208.06 y 100.02 (µg/mL·min^−1^) for the other parameter, with the same order. Regarding the mean residence time (MRT), the values obtained were 40.16 min and 41.39 min for the sphaeralcic acid and the mixture of coumarins (Table 4).

The analysis of the pharmacokinetic behavior of the tomentin and scopoletin mixture and sphaeralcic acid were similar; the compounds conform to the two-compartment model, as indicated by the Information Criteria of Akaike (AIC) and the Schwarz Criterion (SC). The values of AIC and SC (Table 4) are lower for the two-compartment model with respect to the one-compartment model [16]. The adjustment to the two-compartment model is also observed in the behavior of plasma compounds (Figure 3 and Figure 4).

The data were analyzed by the Pk Solver in the one- and two-compartment models. To establish the behavior of the concentration variation of the analyzed compounds, the following functions were used: (one-compartment model) Cp = Ae^−kdt^ + Ce^−kat^, and (two-compartment model) Cp = Ae^−αt^ + Be^−βt^ + Ce^−kdt^. Cp is the plasma concentration (µg/mL); A, B, and C are the plasma concentration (µg/mL) of sphaeralcic acid and coumarins (tomentin and scopoletin) in the central and peripheral compartments, respectively. *k*_a_ is the apparent first-order absorption rate constant. *k*_12_ is the apparent first-order transfer rate constant from the central compartment to the peripheral compartment. *k*_10_ is the apparent first-order elimination rate constant from the central compartment. *k*_21_ is the apparent first-order transfer rate constant from the peripheral compartment to the central compartment. t_1/2_α and t_1/2_β are the absorption half time for the central and peripheral compartment. α and β are the empirical constants corresponding to the coefficients of the exponents of the values of A and B. t_1/2_*k*_a_ is the absorption half time. V/F is the apparent volume of distribution related to the bioavailability of the active substance. T_max_ is the time to maximal concentration. C_max_ is the maximal concentration. CL/F is the apparent clearance related to the bioavailability of the active substance. AUC_0→_∞ and AUC_0–240_ min are the Areas Under the plasma Curve (AUC) from 0 to infinity and from 0 to 240 min, respectively, and MRT is mean residence time. Goodness-of-fit was assessed with Akaike Information Criteria (AIC), Sum of Squares (SS), Schwarz Criteria (SC), and a determination coefficient (*R*^2^). The best model, adjusted according to values of Akaike Information Criteria (AIC), Sum of Squares (SS), Schwarz Criteria (SC), and by determination coefficient (*R*^2^), was the two-compartment model

#### 2.4.2. Pharmacokinetic Intravenous Administration

Table 5 shows the pharmacokinetic parameters of the behavior of the contraction of sphaeralcic acid and the mixture of coumarins, after intravenous administration of the standardized SaTSES fraction. The calculated values for the compounds are contrasted in the table, under two types of distribution, the one-compartment and two-compartment models. The two-compartment model is described and discussed more broadly, because the diagnosis derived from AIC, SC [16], and R^2^ have a better fit for such model. Sphaeralcic acid is more concentrated in the peripheral compartment (B) at 40.46 µg/mL than the central compartment (A) at 18.03 µg/mL, while the opposite is true for the mixture of scopoletin and tomentine, which is more concentrated in (A) 63.57 µg/mL than in (B) 27.86 µg/mL. The apparent first-order elimination rate constant (*k*_10_) was 2.6 times higher for coumarins compared to the same parameter for sphaeralcic acid. Sphaeralcic acid has a transfer rate from the central to the peripheral compartment (*k*_12_) 0.249 min^−1^, which is less than the same parameter for the coumarin mixture that was 0.787 min^−1^. In the opposite direction, the apparent first-order velocity constant from the peripheral compartment to the central compartment (*k*_21_) was 0.624 min^−1^ for sphaeralcic acid and 0.424 min^−1^ for coumarin mixing. For both sphaeralcic acid and the coumarin mixture, there was a shorter average half-life in the central compartment (t_½_α) at 0.781 min and 0.534 min, compared to the average lifetime in the peripheral compartment (t_½_β) at 20.634 min and 16.70 min, respectively. Regarding the initial concentration (C_0_) of sphaeralcic acid and the mixture of scopoletin and tomentine, 58.49 and 91.44 µg/mL, the volume of distribution (V), presenting values of 0.59 and 0.068 (mg)/(µg/mL) in the same order, regarding the clearance (CL), was three times higher for sphaeralcic acid (0.028) compared to the CL value of the coumarin mixture (0.009) with both units of (mg·min)·(µg·mL)^−1^. The areas under the curve (AUC) for sphaeralcic acid and the coumarin mixture were calculated for the defined start interval (0 min) at the end of the evaluation (60 min) and for infinite dilution (0 → ∞), obtaining the following values: 1098.37 and 1277.98 µg/mL·min for the first of the parameters; 665.02 and 720.77 µg/mL·min for the other parameter, in the same order. Regarding the calculation of the entire period of time that elapses in which the total mass of active compounds remain in the system, which was measured with the area under the curve from the initial moment (AUMC), it was more than twice for sphaeralcic acid (35,880.30) compared to the coumarin mixture (16,230.34) measured in µg/mL·min^2^. This determines the value of the mean residence time (MRT), being higher for the sphaeralcic acid mixture and scopoletin/tomentin at 29.29 min and 22.52 min, respectively. Finally, the apparent volume of distribution at steady state (Vss) was calculated, and the value reached by sphaeralcic acid (0.826) was more than four times greater with respect to the coumarin mixture (0.195) quantified in mg/mL µg. As mentioned before, the behavior of both sphaeralcic acid and the mixture of coumarins, in intravenous administration, were better fitted to the two-compartment model, since the diagnostic Akaike Information Criterion (AIC) and Shawn Criterion (SC) parameters were lower for the two-compartment model with the following values: For spaheralcic acid, AIC_2-compartment_ 20.33 < AIC_1-compartment_ 34.13; SC_2-compartments_ 21.12 < 34.52 SC_1-compartment_; for the coumarin mix, the values were: AIC_2-compartment_ 19.49 < AIC_1-compartment_ 28.12; SC_2-compartments_ 20.28 < 28.5 SC_1-compartment_. The above can also be appreciated by the graphic behavior shown in Figure 5 and Figure 6.

The data were analyzed by the Pk Solver in the one- and two-compartment models. To establish the behavior of the concentration variation of the analyzed compounds, the following functions were used: (one-compartment model) Cp = Ae^−kdt^ + Ce^−kat^, and (two-compartment model) Cp = Ae^−αt^ + Be^−βt^ + Ce^−kdt^. Cp is the plasma concentration (µg/mL; A, B, and C are the plasma concentration (µg/mL) of sphaeralcic acid and coumarins (tomentin and scopoletin) in the central and peripheral compartments, respectively; *k*_a_ is the apparent first-order absorption rate constant. *k*_12_ is the apparent first-order transfer rate constant from the central compartment to the peripheral compartment. *k*_10_ is the apparent first-order elimination rate constant from the central compartment. *k*_21_ is the apparent first-order transfer rate constant from the peripheral compartment to the central compartment. t_1/2_α and t_1/2_β are the absorption half time for the central and peripheral compartment. α and β are the empirical constants corresponding to the coefficients of the exponents of the values of A and B. C_0_ is the initial drug concentration at time zero. V is the apparent distribution volume. CL is the apparent total body clearance. AUC_0→_∞ and AUC_0–60_ min are the Areas Under the plasma Curve (AUC) from 0 to infinity and from 0 to 60 min, respectively, and AUMC is the area under the first moment-time curve. MRT is the mean residence time and Vss is the apparent volume of distribution at steady state. Goodness-of-fit was assessed with Akaike Information Criteria (AIC), Sum of Squares (SS), Schwarz Criteria (SC), and a determination coefficient (*R*^2^). The best model, adjusted according to values of Akaike Information Criteria (AIC), Sum of Squares (SS), Schwarz Criteria (SC), and by determination coefficient (*R*^2^), was the two-compartment model.

## 3. Discussion 

In this work, the analytical method was implemented for scopoletin, tomentin, and sphaeralcic acid; to our knowledge, this is the first report of a validated method for tomentin and sphaeralcic acid detection. The dose of fraction for the pharmacocynetic study was chosen according to previous research on active compounds, as the median effective dose, and of detectable concentrations of these compounds in the SaTSS fraction according to the analytical method. When the SaTSS fraction was analyzed, a single peak was observed, consisting of the mixture of tomentin and scopoletin with a rt = 11.457 min, as both compounds have a very similar chemical structures and have very close retention times. Then, the SaTSS fraction administered (400 mg/kg) was standardized in a mixture of tomentin and scopoletin (with 7.1 mg/kg) and sphaeralcic acid (34.6 mg/kg). 

The drug absorption is one of the most important phases of the pharmacokinetic analyses, which defines the behavior of the active compounds concentration in the plasma with respect to time [16]. After oral administration of an SaTSS fraction, a mixture of scopoletin and tomentin and sphaeralcic acid were bioavailable in plasma and products derived from their biotransformation were not detected. The compounds were adjusted to a two-compartment model and they can be considered to be active compounds since no products derived from their biotransformation were detected.

The coumarin mixture and sphaeralcic acid are found more in the central compartment (A) than in the peripheral compartment (B). The first-order absorption rate constant (*k*_a_) for coumarins (0.390 min^−1^) was higher than that of sphaeralcic acid (0.273 min^−1^), indicating that scopoletin and tomentin are absorbed faster than the sphaeralcic acid. The apparent first-order elimination rate of the central compartment (*k*_10_) depends on the amount of compound present in the central compartment; this constant is higher for sphaeralcic acid (0.127 min^−1^) than for the coumarin mixture (0.085 min^−1^), and this correlates with a higher sphaeralcic acid concentration in the central compartment than in the peripheral one. The apparent first-order transfer rate (*k*_12_) from the central to the peripheral compartment was 57% greater for the mixture of coumarins than for sphaeralcic acid. This correlated with the return rate values from the peripheral compartment to the central compartment (*k*_21_), which are also superior for the mixture of coumarins. Therefore, for both compounds, the average lifetime in the peripheral compartment t_1/2_β is greater than the average lifetime in the central compartment t_1/2_α. 

When the parameters of drug distribution, such as the apparent volume of distribution with respect to bioavailability (V/F), where the administration was oral, were compared, the value for coumarins was 0.802 mg·(μg/mL)^−1^, which was less than the value obtained with sphaeralcic acid mg·(μg/mL)^−1^. Thus, for the total apparent clearance regarding bioavailability (CL/F) after oral administration of the drug, the value for sphaeralcic acid was two times higher than what was calculated for coumarins. Sphaeralcic acid reaches a C_max_ of 10.45 μg/mL at 4.00 min, and the coumarins have a C_max_ of 3.77 μg/mL at 3.23 min. The latter was measured by the AUC of plasma concentrations (AUC_0__,__∞_ and AUC_0__,__240_), reflecting the total amount of the drug reaching the systemic circulation, the and Main Residence Time (MRT) was very similar for both sphaeralcic (40.17 min) acid and the coumarin mix (41.40 min). 

In intravenous administration, spaheralcic acid was found to be more concentrated in the peripheral compartment [B] compared to the central compartment [A]. The opposite happened with the mixture of scopoletin and tomentin, which indicated that sphaericalcic acid, administered intravenously, reached the central compartment. However, due to its chemical structure or cellular transport, it could reach the peripheral compartment more efficiently than the mixture of coumarins. This value was relevant because the compounds had a pharmacological effect on the joints and should reach the peripheral compartment more easily. Because the coumarin mixtures had a higher concentration in the central compartment [A], compared to sphaeralcic acid, in intravenous administration, the elimination constant *k*_10_ was 2.6 times higher than the same parameter in sphaeralacic acid, since this value depends on the concentration in the central compartment [A]. In the same way, as the value for [A] was higher, the transfer rate from the central compartment to the peripheral *k*_12_ was also higher than for sphaeralcic acid. This was contrary to what was observed in the transfer rate from the peripheral compartment to the central compartment *k*_21_, due to the fact that sphaeralcic acid is more concentrated in the peripheral compartment [B]. This correlated with the half-life time in the central compartment t_½α_ for coumarins and sphaeralacic acid, being higher for the former, and in the same sense for the half-life time t_½β_ in the peripheral compartment. The volume of distribution (V) was higher for sphaericalcic acid compared to the V value of the mixture of coumarins. The same trend was observed when the active fraction was administered orally. This revealed a strong tendency for sphaericalcic acid to distribute towards the peripheral compartment, both in intravenous and oral administration. This correlated perfectly with the result that sphaeralcic acid was more concentrated in the peripheral compartment [B] than in the central compartment [A]. With respect to the AUC and AUMC values, when calculating the relationship of what happened with sphaeralcic acid with respect to the coumarin mixture, a relationship of between 1.6 and 1.7 of the first was observed, with respect to the coumarin mixture. Therefore, it can be assumed that sphaeralcic acid had greater bioavailability and had a greater capacity to reach the systemic circulation. Sphaericalcic acid presented a higher value of the concentration of the apparent volume of distribution at steady state (Vss). It is known that Vss indicates that the compounds found in the body are not so dissolved in the body fluid, but rather that these compounds can be bound to plasma proteins and other macromolecules and tissues. That is, they are not uniformly distributed, and the value does not correspond to a real physiological volume, confirming the fact that sphaeralcic acid reaches the peripheral compartment in a proportion of 4.2 times that the mixture of coumarins. One of the main contributions that can be obtained from the pharmacokinetic test was from the intravenous administration of both spheric acid and the coumarin mixture, establishing parameter F, that is, the absorption value, which was calculated in relation to the V / F and CL / F values during oral administration of the extract with the SaTSS fraction. The F parameter presented the following values: Between 16 and 45% for spherical acid and between 8 and 13% for coumarins. Finally, in intravenous administration, the two-compartment model was the one that best adjusted to the distribution process, which was defined from the diagnosis of AIC, SC SS, and R^2^ [17,18,19].

With these analyses, it was possible to define that, after their absorption, coumarins and sphaeralcic acid are distributed in the organism. This hypothesis is supported by previous reports about pharmacokinetic parameters of elimination of the SaTSS fraction by oral administration at the same doses (400 mg/kg), where sphaeralcic acid and coumarins mixture were identified in feces and urine; the mixture of scopoletin and tomentine were eliminated by the renal route and the elimination of sphaeralcic acid was by the enterohepatic route [20].

*Sphaeralce angustifolia* is a medicinal plant with active compounds that have pre-clinical studies of safety and pharmacological activity, compound stability, and pharmacokinetic analysis of the absorption and elimination necessary for phytomedicine registration and to provide a quality product to the consumer.

## 4. Material and Methods

### 4.1. Cell Suspension Culture

*Sphaeralcea angustifolia* cell suspension in batch cultures was cultivated with an inoculum of 5% of fresh biomass in 500 mL of liquid MS medium [21] with 2.74 mM of total nitrate supplied with 1.0 mg/L of naphthaleneacetic acid, 0.1 mg/L of kinetin, 30.0 g/L of sucrose, adjusted to pH 5.7, previously autoclaved to 1 kg/cm^2^ for 18 min at 120 °C. Flasks of cell suspensions were placed in an orbital shaker at 110 rpm (New Brunswick Scientific Co., Edison, NJ, USA) and incubated at 26 ± 2 °C under 50 µM/m/s light intensity with warm-white fluorescent light and a photoperiod of 16 h of light and 8 h of darkness. Cultures were arrested on day 16 of culture to obtain the biomass [14,22].

### 4.2. Extraction and Purification Process of Sphaeralcea Angustifolia Cell Suspension

Cells cultivated in suspension were filtered and the biomasses were pooled and dried at room temperature. Then, dry biomass (100 g) was extracted three times by maceration at room temperature with a mixture of grade-reactive solvent (CH_2_Cl_2_:CH_3_OH 9:1; Merck, Mexico City, Mexico) at a ratio of 1:20 (*w/v*) at 24 h for each one. The extracts were filtered, pooled, and concentrated to dryness under reduced pressure in a rotaevaporator (Büchi R-124. Postfach, Switzerland). This procedure was performed several times.

### 4.3. Preparation of the Sates Active Fraction 

The CH_2_Cl_2_:CH_3_OH extract (10 g) was fractionated by silica gel column chromatography (9 × 28 cm, 70–230 mesh; Merck) employing a gradient system of *N*-hexane:ethyl-acetate:methanol grade-reactive solvents (Merck, Kenilworth, NJ, USA) with 5–10% of polarity increments [14]. Aliquots of 500 mL were collected and concentrated to dryness under reduced pressure; samples with compounds with a TLC profile and fluorescence similar to tomentin, scopoletin (50:50), and sphaeralcic acid (60:40) were integrated into SaTES fraction, which was then analyzed by HPLC and standardized based on the content of coumarin mixture (tomentin and scopoletin) and sphaeralcic acid. This procedure was performed several times until the necessary amount of tomentin, sphaeralcic acid, and SaTES fraction were obtained to perform the analytical method validation and pharmacokinetic experiments.

### 4.4. Isolation of Tomentin and Sphaeralcic Acid 

Parallel to and independently from *N*-hexane:ethyl-acetate:methanol fractions, tomentin (50:50) and sphaeralcic acid (60:40) were purified through an open silica gel RP-18 column (1.5 × 28 cm, 40–63 mesh; Merck) with an H_2_O:CH_3_CN elution system (high purity solvents; Merck, Kenilworth, New Jersey, USA) with an increasing polarity of 10%. Aliquots of 20 mL were obtained, tomentin was isolated from pooled sub-fraction of 80:20-H_2_O:CH_3_CN and sphaeralcic acid from 50:50-H_2_O:CH_3_CN. The compounds were analyzed by HPLC, and their purity was confirmed by comparing their retention times and absorption spectra [14,15,17]. This procedure was performed several times until the necessary amount of tomentin and sphaeralcic acid was obtained.

### 4.5. HPLC Calibration Curves of Tomentin, Scopoletin, and Sphaeralcic Acid 

Scopoletin (99% purity, Sigma-Aldrich, Mexico City, Mexico), tomentin (98% purity), and sphaeralcic acid (99% purity) calibration curves were performed using the HPLC system from a 1.0 mg/mL solution in high-purity methanol (Merck, Kenilworth, NJ, USA). Six serial dilutions were performed at concentrations of 1.25–20 µg/mL for scopoletin, 0.625–20 µg/mL for tomentin, and 2.5–40 µg/mL for sphaeralcic acid. The chromatograms were analyzed at λ = 343 nm for tomentin and scopoletin, and at λ = 357 nm for sphaeralcic acid. Each calibration curve was constructed by plotting the peak area ratio of the compound (y) versus the analyte concentrations (x). Calibration curves of tomentin, scopoletin, and sphaeralcic acid were fitted using a linear square model (y) = m (x) + b using Microsoft Office Excel software 2010 with correlation values of ≥ 0.9995.

### 4.6. Conditions of HPLC Analysis 

Analyses of HPLC were carried out in a Waters system (2695 Separation module) coupled to a diode array detector (2996) with a 190–600-nm detection range and operated by the Manager Millennium software system (Empower 1; Waters Corporation, Mexico City, Mexico). Separations were performed in a Spherisorb-ODS RP-18 column (250 × 4.6 mm, 5 µm; Waters Corporation, Mexico) employing a constant temperature of 25 °C during the analyses. Samples (20 µL) were eluted at a 1.0 mL/min flow rate with a gradient of mobile phases of (A) high-purity H_2_O with trifluoroacetic acid to 0.5% *v/v* (TFA, Sigma-Aldrich, Mexico City, Mexico) and (B) high purity CH_3_CN, and compounds were detected by monitoring absorbance for tomentin and scopoletin at λ = 343 nm and for sphaeralcic acid at λ = 357 nm. The mobile phase was started with water (100%) and was maintained for 1 min, then solvent B was gradually incorporated at 5% (at 2 min), at 30% (at 4 min), and at 50% (at 16 min). During the next 4 min, solvent B was increased to 100%, and this proportion was maintained for 5 min. Finally, the following 3 min were utilized to return the mobile phase to the initial condition. The chromatographic method had a 25 min run time. Identification of scopoletin and tomentin as well as sphaeralcic acid was performed by comparing their retention times (tomentin—11.288 min, scopoletin—11.349 min, coumarin mixure—11.457 min, and sphaeralcic acid—23.319 min) and absorbance spectra [14,15,17].

### 4.7. Standards and Internal Standard Stock Solutions for Chromatographic Profiling

The standard solutions of tomentin, scopoletin, and sphaeralcic acid were prepared at a nominal concentration of 1 mg/mL of high purity methanol (Merck, Kenilworth, NJ, USA); subsequently, serial dilutions for each compound were made to obtain different concentrations. The internal standard dopamine was prepared at a nominal concentration of 2 mg/mL of high-purity methanol; this was added to tomentin, scopoletin, and sphaeralcic acid solutions with a final concentration of 10 µg/mL.

### 4.8. Animals 

ICR mice, the strain named by the initial letters of the Institute of Cancer Research in the American United States, weighing 30–35 g, were used (Envigo, Ciudad de México, Mexico) and kept in groups with similar treatment of 10 animals per cage (19 groups, 190 mice in total) under laboratory conditions at 25 °C, with a 12 h light/dark cycle, and water and food (pellets from Harlan Rodent Lab Diet) with ad libitum access. The adaptation time to the laboratory conditions before the experiments was 3 weeks. All of the studies were implemented in accordance with the Mexican Official Regulation NOM-062-ZOO-1999 [23]. The ethical use of animals was approved through the Local Committee for Research in Health, and Ethics from Instituto Mexicano del Seguro Social (IMSS), who assigned the registration number R-2016-1702-10 to the protocol. To obtain consistent data, a minimal number of animals and time of observation were used.

### 4.9. Obtaining Mouse Plasma 

The mice were previously anesthetized with sodium pentobarbital (PiSA Agropecuaria), then retro-orbital sinus blood was obtained using heparinized capillaries and distributed to heparinized tubes, and tubes were centrifuged at 3500 g for 7 min. The plasma obtained was transferred to new tubes and stored at −4 °C until use.

### 4.10. HPLC Calibration Curves of Tomentin, Scopoletin, and Sphaeralcic Acid In Plasma 

Plasma samples were prepared with tomentin, scopoletin, and sphaeralcic acid according to the standards presented previously by adding the stock solutions to the mice plasma. Calibration curves for plasma samples were prepared throughout a linear range of 0.625, 1.25, 2.5, 5, 10, and 20 µg/mL for tomentin, 1.25, 2.5, 5, 10, and 20 µg/mL for scopoletin, and 2.5, 5, 10, 20, and 40 µg/mL for sphaeralcic acid. Each calibration curve was prepared with five or six concentrations and compared against a double blank sample with and without internal standards.

### 4.11. Extraction of Active Compounds in Plasma 

Plasma samples were extracted with CH_3_CN and TFA (*w/v*) 20% (1:1), then vials were centrifuged at 14,000 g for 10 min and the supernatant was transferred to another vial and left to dry at room temperature. The precipitate was resuspended with 1 mL of high-purity methanol (Merk, México) and filtered through Teflon membrane (13 mm, 0.45 µm, Life Science). Each filtered sample was injected into the HPLC equipment, and the concentrations of tomentin and scopoletin at λ = 343 nm and of sphaeralcic acid at λ = 357 nm were obtained by comparison with pre-built calibration curves.

### 4.12. Method Validation 

Validation of analytical methods was performed according to U.S. Food and Drug Administration (FDA) guidelines [24].

#### 4.12.1. Linearity and Sensitivity Test 

To evaluate the linearity of standard calibration curves, determinations of active compounds in plasma samples were accomplished on 6 independent days using freshly prepared samples. Calibration curves for plasma samples were prepared throughout a linear range of 0.625–20 µg/mL for tomentin, 1.25–20 µg/mL for scopoletin, and 2.5 to 40 µg/mL for sphaeralcic acid. Each calibration curve was prepared with five concentrations and compared against a double- blank sample with and without internal standards. Each calibration curve was constructed by plotting the peak area ratio of the compound (y) versus the analyte concentrations (x). Curves were fitted using a linear least-square regression model (y) = m(x) + b utilizing Microsoft Office Excel 2010 software. The resulting m and b parameters were employed to determine the back-calculated concentrations, which were evaluated statistically. All calibration curves of tomentin, scopoletin, and sphaeralcic acid were created prior to the experiments with linear correlation values of ≥0.9995.

#### 4.12.2. Recovery (Extraction Efficiency) 

The extraction efficiency of tomentin, scopoletin, and sphaeralcic acid was determined by analyzing a series of replicates (*n* = 5) of Quality Control (QC) samples of 0.625–20 µg/mL for tomentin, of 1.25–20 µg/mL for scopoletin, and of 2.5–40 µg/mL for sphaeralcic acid in mouse plasma. Recovery was calculated by comparing the peak areas of tomentin, scopoletin, and sphaeralcic acid, which were added into the blank samples and extracted using the protein precipitation procedure, with those obtained from tomentin, scopoletin, and sphaeralcic acid that were added directly into the post-protein precipitation solvent at QC concentration levels.

#### 4.12.3. Specificity Test 

The specificity test was defined by two conditions: A non-interference term when the tomentin, scopoletin, and sphaeralcic acid were not retained by the endogenous components of plasma, and second, no cross-interference among tomentin, scopoletin, and sphaeralcic acid with the internal standard using the proposed extraction procedure and HPLC conditions. Three different plasma samples were used as blanks (plasma free of tomentin, scopoletin, and sphaeralcic acid) and were extracted and analyzed by HPLC with and without internal standard to assess the specificity of the method.

#### 4.12.4. Accuracy and Precision Test 

Intra- and inter-assay accuracies were expressed as the percentage of difference between the measured concentration and the nominal concentration. Intra-assay precision and accuracy were calculated using replicate determinations (*n* = 6) for each concentration of tomentin, scopoletin, and sphaeralcic acid that were added to the plasma samples during a single analytical run. Inter-assay precision and accuracy were calculated using replicate determinations (*n* = 6) for each concentration of tomentin, scopoletin, and sphaeralcic acid, and these were performed on 6 separate days. Accuracy was calculated utilizing the following equation: (%Bias) = [(C_obs_ − C_nom_)/C_nom_] × 100. Precision was calculated from the observed concentrations as follows: RSD = [Standard Deviation (SD)/C_obs_] × 100. Accuracy (Bias) and precision (RSD) values were within ± 15%, covering a range of actual experimental concentrations that were considered acceptable.

#### 4.12.5. Stability Study 

The stability of tomentin, scopoletin, and sphaeralcic acid in mouse plasma was assessed by analyzing the replicates (*n* = 5) of the QC samples at three different concentrations (2.5, 5, and 10 µg/mL). The investigation presented here expected manipulation conditions during all of the sample storage and process periods, which included the stability data from the freeze/defrost, bench-top, autosampler, and long-term stability tests. For all stability studies, fresh QC samples were evaluated by using a freshly prepared standard curve for the measurements. The analyzed conditions were 0, 8, and 24 h at room temperature, 8 h at 4 °C, and 30 days at −70 °C. The concentrations obtained from all of the stability studies were compared with the fresh QC samples, and the percentage of concentration deviation was calculated. Tomentin, scopoletin, and sphaeralcic acid were considered stable in the mouse plasma when the concentration difference between the freshly prepared samples and the stability samples was less than 15%.

### 4.13. Pharmacokinetic Analysis 

To evaluate the suitability of the assay for the pharmacokinetic studies, 400 mg/kg of the active SaTSS fraction standardized in a mixture of tomentin and scopoletin (7.1 mg/400 mg), and sphaeralcic acid (34.6 mg/400 mg) was administered orally or intravenously to the mice. The dose of SaTSS fraction was chosen based on previous research of active compounds, as the median efective dose of each compound and detectable concentrations of them in the fraction. For oral pharmacokinetics, ten animals were used in each group at different times (0, 1.3, 3.3, 5, 10, 20, 40, 60, 80, 120, and 240 min). For intravenous pharmacokinetics, three animals were used in each group at different times (2, 4, 6, 8, 10, 15, 20, 30, and 60 min) Subsequently, the mice were anesthetized, retro-orbital sinus blood was obtained, plasma samples were extracted with CH_3_CN and TFA (*w/v*) 20% (1:1), and the extracts were analyzed by HPLC for coumarin (tomentin and scopoletin) and sphaeralcic acid quantification. Pharmacokinetic calculations were performed using the observed data. All data were subsequently processed using the PKSolver add-in program for Microsoft Excel written in Visual Basic for Applications. All values obtained were expressed in mean ± Standard Deviation (SD). For the pharmacokinetic assay, the selection was made either from a one-compartment or from a two-compartment model, with the use of both the Akaike Information Criterion (AIC) and the Schwarz Criterion (SC). Using these two criteria, it is possible to select which model is more suited to being adjusted, at the point at which it reaches the lowest values of the AIC or SC criteria, meaning that the chosen model is more parsimonious (fewer parameters required) and best fits the data (low error prediction) [18].

## 5. Conclusions

A reliable analytical method was developed for anti-osteoarthritic compounds tomentin, scopoletin, and sphaeralcic acid, which was useful in their pharmacokinetic evaluation. The compounds were adjusted to the two-compartment model and are bioavailable in plasma; therefore, they are considered drugs with potential osteoantiarthritic activity because no products derived from their biotransformation were detected. The information provided will allow the design of a phytomedicine for oral administration, effective and safe, useful for the treatment of osteoarthritis. Finally, the possibility of designing a pharmaceutical formulation with purified compounds that satisfy with current regulations for intra-articular administration products is opened.

## Figures and Tables

**Table 1:** Tomentin, scopoletin, and sphaeralcic acid by intra- and inter-assays in mice measured in plasma (matrix), and precision and accuracy determination.

Compound	Test	Concentration Nominal µg/mL)	Measured Concentration µg/mL± SD	Accuracy (% Bias)	Precision (% RDS)
Blank plasma	Intra-assay	0	0	0	0
	Inter-assay	0	0	0	0
Tomentin	Intra-assay	2.5	2.41 ± 0.07	−3.4	2.8
		5	4.94 ± 0.06	−1.2	1.1
		10	9.95 ± 0.13	−0.5	1.3
		20	19.94 ± 0.22	−0.3	1.1
	Inter-assay	2.5	2.39 ± 0.09	−4.6	3.6
		5	4.93 ± 0.11	−1.5	2.3
		10	9.96 ± 0.30	−0.4	3.0
		20	19.77 ± 0.19	−1.1	1.0
Scopoletin	Intra- assay	2.5	2.33 ± 0.08	−6.6	3.5
		5	5.06 ± 0.06	1.1	1.1
		10	9.98 ± 1.27	−0.2	2.7
		20	19.26 ± 0.25	−3.7	1.3
	Inter- assay	2.5	2.20 ± 0.04	−12.0	1.9
		5	5.08 ± 0.05	1.6	0.9
		10	9.78 ± 0.14	−2.2	1.4
		20	19.38 ± 0.45	−3.1	2.3
Sphaeralcic acid	Intra- assay	2.5	2.33 ± 0.17	−6.8	7.2
		5	5.06 ± 0.09	1.3	1.7
		10	9.96 ± 0.12	−0.4	1.2
		20	20.23 ± 1.31	1.2	6.5
		40	39.52 ± 1.19	−1.2	3.0
	Inter- assay	2.5	2.18 ± 0.13	−13.0	6.1
		5	4.91 ± 0.13	−1.9	2.7
		10	9.90 ± 0.7	−1.0	0.7
		20	19.91 ± 0.19	−0.5	0.9
		40	39.36 ± 0.75	−1.6	1.9

Mean ± Standard deviance of the Mean; *n* = 6.

**Table 2:** Stability profile of active compounds tomentin, scopoletin, and sphaeralcic acid contained in the SaTSS fraction from *Sphaeralceae angustifolia* cell suspension in mice plasma (matrix) under different handling conditions.

Condition	Compound	Nominal Concentration (μg/mL)	Observed Concentration (μg/mL)			Accuracy Bias (%)			RSD (%)		
			0 h	8 h	24 h	0 h	8 h	24 h	0 h	8 h	24 h
Room temperature	Tomentin	2.5	2.39 ± 0.09	2.31 ± 0.08	2.35 ± 0.09	−4.59	−7.60	−5.93	3.57	3.30	3.90
		5	4.93 ± 0.11	4.96 ± 0.14	4.91 ± 0.17	−1.48	−0.86	−1.73	2.31	2.88	3.42
		10	9.96 ± 0.30	9.84 ± 0.40	9.55 ± 0.19	−0.41	−1.64	−4.52	2.97	4.10	1.99
	Scopoletin	2.5	2.49 ± 0.12	2.38 ± 0.08	2.38 ± 0.08	−0.48	−4.63	−4.63	4.83	3.15	3.15
		5	5.02 ± 0.08	4.99 ± 0.14	5.02 ± 0.10	0.39	−0.17	0.43	1.53	2.82	2.07
		10	10.00 ± 0.37	9.80 ± 0.40	9.34 ± 0.27	0.04	−2.02	−6.61	3.65	4.05	2.89
	Sphaeralcic acid	2.5	2.48 ± 0.03	2.25 ± 0.13	2.27 ± 0.11	−0.60	−9.98	−9.03	1.10	5.97	5.01
		5	5.01 ± 0.06	4.59 ± 0.06	4.56 ± 0.10	0.17	−8.10	−8.73	1.11	1.27	2.09
		10	9.95 ± 0.11	9.35 ± 0.11	9.26 ± 0.07	−0.49	−6.48	−7.37	1.12	1.21	0.75
Storage and stability		Nominal concentration (μg/mL)	Auto sampler (4 °C, 8 h)	Long-term (−70 °C, 1 month)	Autosampler (4 °C, 8 h)	Long-term (−70 °C, 1 month)			Auto sampler (4 °C, 8 h)	Long-term (−70 °C, 1 month)	
	Tomentin	2.5	2.43 ± 0.11	2.31 ± 0.09	−2.64	−7.61			4.47	3.92	
		5	4.92 ± 0.14	4.85 ± 0.18	−1.65	−2.90			2.82	3.75	
		10	9.93 ± 0.62	9.63 ± 0.65	−0.65	−3.74			6.28	6.71	
	Scopoletin	2.5	2.52 ± 0.09	2.32 ± 0.08	0.75	−7.05			3.65	3.57	
		5	4.95 ± 0.14	4.89 ± 0.17	−0.94	−2.27			2.76	3.48	
		10	9.90 ± 0.61	9.95 ± 0.38	−1.05	−0.46			6.21	3.79	
	Sphaeralcic acid	2.5	2.40 ± 0.04	2.38 ± 0.08	−3.89	−4.82			1.82	3.39	
		5	4.92 ± 0.08	4.87 ± 0.10	−1.69	−2.61			1.61	2.10	
		10	9.67 ± 0.12	9.65 ± 0.08	−3.34	−3.50			1.22	0.79	

**Table 3:** Variation of coumarins (tomentin and scopoletin) and sphaeralcic acid concentrations in plasma during the initial 4 h after oral administration and in the first hour after intravenous administration, of a single dose (400 mg/kg) of the SaTES standardized fraction in ICR mice.

Oral Administration Plasma Concentration			Intravenous Administration Plasma Concentration		
Time (min)	Coumarins (Tomentin and Scopoletin) (μg/mL)	Sphaeralcic Acid (μg/mL)	Time (min)	Coumarins (Tomentin and Scopoletin) (μg/mL)	Sphaeralcic Acid (μg/mL)
1.3	2.37 ± 0.05	5.04 ± 0.05	2.0	30.5 ± 0.12	40.88 ± 0.21
3.3	4.41 ± 0.07	9.92 ± 0.41	4.0	23.54 ± 0.47	36.28 ± 0.11
5	3.21 ± 0.15	12.84 ± 0.54	6.0	22.11 ± 0.85	33.90 ± 0.24
10	1.80 ± 0.09	4.03 ± 0.11	8.0	20.97 ± 0.40	31.78 ± 0.75
20	1.38 ± 0.05	2.77 ± 0.08	10	18.49 ± 0.41	28.71 ± 0.65
40	0.95 ± 0.03	1.38 ± 0.12	15	15.33 ± 0.18	24.43 ± 0.33
60	0.48 ± 0.01	0. 69 ± 0.03	20	12.76 ± 0.19	21.16 ± 0.51
80	0.12 ± 0.01	0.17 ± 0.03	30	8.46 ± 0.21	15.79 ± 0.57
120	0.00 ± 0.0	0.00 ± 0.0	60	0.68 ± 0.04	3.81 ± 0.09
240	0.00 ± 0.0	0.00 ± 0.0			

Mean ± Standard Deviation (SD) of the Mean; *n* = 10 for oral administration and *n* = 3 for intravenous administration.

**Table 4:** Pharmacokinetic parameters after oral administration of the coumarins (tomentin and scopoletin) and sphaeralcic acid present in the SaTSS standardized fraction (400 mg/kg) of *Sphaeralcea angustifolia* in the plasma of ICR mice; *n* = 10. The analytes fit into the two-compartment model.

Pharmacokinetic Parameters	Sphaeralcic Acid	Coumarin (Tomentin and Scopoletin)		Units
A	413.646	71.468		µg/mL
B	1.845	1.791		µg/mL
*k*_a_	0.273	0.390		1/min
*k*_10_	0.127	0.085		1/min
*k*_12_	0.114	0.201		1/min
*k*_21_	0.031	0.086		1/min
t_1/2_α	2.698	1.963		min
t_1/2_β	44.653	32.946		min
t_1/2_*k*a	2.537	1.774		min
α	0.256	0.353		1/min
β	0.015	0.021		1/min
V/F	1.309	0.802		(mg)/(µg/mL)
CL/F	0.166	0.068		(mg)/(µg/mL)/min
T_max_	4.004	3.234		min
C_max_	10.447	3.775		µg/mL
AUC _0__→__240_	173.724	84.198		µg/mL·min
AUC _0__→_∞	208.058	100.020		µg/mL·min
MRT	40.167	41.397		min
	**Diagnostics**			
Statistical criteria	Sphaeralcic acid		Coumarin (Tomentin and scopoletin)	
	One-compartment model	Two-compartment model	One-compartment model	Two-compartment model
SS	18.469	15.62	1.918	0.835
*R* ^2^	0.941	0.950	0.953	0.979
AIC	37.739	32.245	11.862	8.379
SC	35.726	32.836	12.453	9.365

**Table 5:** Pharmacokinetic parameters after intravenous administration of the coumarin (tomentin and scopoletin) and sphaeralcic acid present in the SaTES standardized fraction (400 mg/kg) of *Sphaeralcea angustifolia* in the plasma of ICR mice; *n* = 3. The analysis fit into the one and two compartment model.

	Sphaeralcic Acid		Coumarin (Tomentin and Scopoletin)			
Pharmacokinetic Parameters	One-Compartment Model	Two-Compartment Model	One-Compartment Model		Two-Compartment Model	Units
A	------	18.027	-------		63.57	µg/mL
B	------	40.461	------		27.86	µg/mL
*k*_10_	0.032	0.048	0.047		0.126	1/min
*k*_12_	------	0.249	------		0.787	1/min
*k*_21_	------	0.624	------		0.424	1/min
t_1/2_α	------	0.781	------		0.534	min
t_1/2_β	------	20.634	------		16.70	min
t_1/2_	21.245	------	14.641		-------	min
α	-------	0.88	-------		1.297	1/min
β	-------	0.034	-------		0.041	1/min
C_0_	41.726	58.489	30.773		91.438	µg/mL
V	0.828	0.591	0.220		0.068	(mg)/(µg/mL)
CL	0.027	0.028	0.010		0.009	(mg)/(µg/mL)/min
AUC_0-60_	1098.375	1064.322	612.085		665.022	µg/mL*min
AUC_0-inf_ ∞	1278.978	1224.828	650.047		720.770	µg/mL*min
AUMC	39,202.118	35,880.304	13,731.187		16,230.343	µg/mL*min^2^
MRT	30.651	29.294	21.123		22.518	Min
Vss	0.828	0.826	0.220		0.195	mg/(µg/mL)
	**Diagnostics**					
	Sphaeralcic acid			Coumarin (Tomentin and scopoletin)		
Statistical criteria	One-compartment model	Two-compartment model		One-compartment model		Two-compartment model
SS	28.44	3.937		14.58		3.587
*R* ^2^	0.996	0.999		0.995		0.999
AIC	34.13	20.333		28.116		19.497
SC	34.52	21.122		28.511		20.285

## Data Availability

Data is contained within the article and supplementary material.

## Supplementary Materials

The following are available online at https://www.mdpi.com/article/10.3390/ph14070610/s1, Table S1. Parameters of limit of detection (LOD) and limit of quantification (LOQ) calculated. Table S2. Recovery capacity of active compounds from plasma extracted in 5 different days.
