# Supplementary material for: Pharmacokinetic Study of Anti-osteoarthritic Compounds of a Standardized Fraction from Sphaeralcea Angustifolia
Source: Pharmaceuticals (Basel). 2021 Jun 25;14(7):610. doi: 10.3390/ph14070610 (PMC8308911; doi:10.3390/ph14070610)
Supplement: Supplementary file 1 [file pharmaceuticals-14-00610-s001.zip › pharmaceuticals-1247056-supplementary.pdf]

pharmaceuticals

Article

Pharmacokinetic study of anti-osteoarthritic compounds of a standardized fraction from *Sphaeralcea angustifolia*

Jade Serrano-Román[1-2], Pilar Nicasio-Torres[1,*], Elizabeth Hernández-Pérez[3] and Enrique Jiménez-Ferrer [1,*]

[1] Centro de Investigación Biomédica del Sur (CIBIS), Instituto Mexicano del Seguro Social (IMSS), Argentina 1, Centro, CP 62790 Xochitepec, Morelos, Mexico pisaliva@yahoo.com.mx (MPN-T): enriqueferrer_mx@yahoo.com (EJ-F)

[2] Doctorado en Ciencias Biológicas y de la Salud, División de Ciencias Biológicas y de la Salud, Universidad Autónoma Metropolitana Unidad Iztapalapa (UAM-Iztapalapa). San Rafael Atlixco 186, Vicentina, Iztapalapa, CP 09340 Ciudad de México (CDMX), México. jadita_9@hotmail.com (JS-R).

[3] División de Ciencias Biológicas y de la Salud, Universidad Autónoma Metropolitana Unidad Iztapalapa (UAM-Iztapalapa), San Rafael Atlixco 186, Vicentina, Iztapalapa, CP 09340 CDMX, México. sila@xanum.uam.mx (EH-P).

Table S1. Parameters of limit of detection (LOD) and limit of quantification (LOQ) calculated.

Compound	Slope of calibration curve (S)	Standard deviation (σ)	Detection limit (LOD)= 3.3σ/S	Quantitation limit (LOQ)=10σ/S
Tomentin	162940	3385.90	0.068	0.207
Scopoletin	165407	6976.26	0.139	0.421
Sphaeralcic acid	7381.9	133.30	0.059	0.180

Values *n*=6.

Table S2. Recovery capacity of active compounds from plasma extracted in 5 different days.

Compound	Nominal concentration (μg/mL)	Peak area of compound in solution	Peak area of compound in plasma	Recovery (%)	Mean (%)±SD	RSD (%)
**Tomentin**	**2.5**	362298	360010	99.4	99.4 ± 0.40	0.40
		370018	369902	100.0		
		376789	372806	98.9		
		367656	364397	99.1		
		362132	360406	99.5		
	**5**	846586	811858	95.9	96.3 ± 2.44	2.53
		829876	799616	96.4		
		859980	800692	93.1		
		840096	808335	96.2		
		824215	823877	100.0		
	**10**	1699392	1568996	92.3	96.7 ± 2.99	3.09
		1679123	1626145	96.8		
		1652019	1650051	99.9		
		1640822	1622143	98.9		
		1680087	1602613	95.4		
	**20**	3131575	3109042	99.3	98.2 ± 1.22	1.24
		3279579	3265492	99.6		
		3300225	3200110	97.0		
		3198001	3103140	97.0		
		3311856	3248992	98.1		
**Scopoletin**	**2.5**	350166	338010	96.5	97.4 ± 2.00	2.06
		365288	348912	95.5		
		358065	342806	95.7		
		355689	354397	99.6		
		354537	352406	99.4		
	**5**	835755	811858	97.1	99.2 ± 1.26	1.27
		829366	829616	100		
		820465	820662	100		
		828338	828335	100		
		838300	828877	98.9		
	**10**	1584170	1568996	99.0	97.3 ± 1.92	1.97
		1681460	1626145	96.7		
		1669683	1596451	95.6		
		1699245	1622143	95.5		
		1599539	1592613	99.6		
	**20**	3219863	3122042	98.8	98.1 ± 1.63	1.66
		3231392	3165492	98.0		

		3159439	3089110	98.1		
		3454103	3303140	95.6		
		3194902	3194881	100		
**Sphaeral-cic acid**	**5**	38974	38121	97.81	97.4 ± 1.48	1.52
		39033	37286	95.52		
		37454	36048	96.25		
		39049	38557	98.74		
		38391	37926	98.79		
	**10**	75604	75066	99.3	99.6 ± 0 35	0.35
		74391	74197	99.7		
		74276	74202	99.9		
		75007	74955	99.9		
		74503	73900	99.2		
	**20**	152030	149335	98.2	99.0±0.80	0.80
		149202	148925	99.8		
		149976	149296	99.5		
		150973	148029	98.0		
		147103	146033	99.3		
	**40**	289536	287123	99.2	98.8±1.03	1.04
		299976	296883	99.0		
		299264	298637	99.8		
		298727	289892	97.0		
		290051	286889	98.9		

Mean ± Standard deviance of the Mean; *n* =5.
